# The Role of Early Pregnancy Maternal Selenium Levels on the Risk for Small-for-Gestational Age Newborns

**DOI:** 10.3390/nu11102298

**Published:** 2019-09-26

**Authors:** Małgorzata Lewandowska, Stefan Sajdak, Jan Lubiński

**Affiliations:** 1 Division of Gynecological Surgery, Poznań University of Medical Sciences, 60-535 Poznań, Poland; ssajdak@ump.edu.pl; 2 Department of Genetics and Pathology, International Hereditary Cancer Center, Pomeranian Medical University, 71-252 Szczecin, Poland; lubinski@pum.edu.pl

**Keywords:** selenium, fetus, weight, newborn, SGA, pregnancy, AGA, microelement, trace element

## Abstract

It has not yet been established, whether or not the maternal serum selenium (Se) in early pregnancy may be a risk marker of small-for-gestational age (SGA) birth weight. Selenium is important for human health and is involved in oxidative balance, a key element in the development of the placenta and fetus. This innovative study was nested in a prospective cohort of 750 women recruited in the 10–14th week of a single pregnancy, all of whom were healthy during recruitment. We examined mothers delivering SGA infants (with birth weight <10th percentile) (*n* = 48) and matched mothers delivering appropriate-for-gestational age (AGA) infants (between 10–90th percentile) (*n* = 192). We measured the maternal microelement concentrations in the serum from the 10–14th gestational week, using the inductively coupled plasma mass spectrometry (ICP-MS). The odds ratios of SGA (and 95% confidence intervals) were assessed in logistic regression. The mean maternal Se concentrations were lower in mothers in the SGA group compared to the AGA group (59.60 vs. 62.54 µg/L; *p* = 0.020). Women in the lowest Q_1_ quartile of Se (≤56.60 µg/L) have about three times higher risk of SGA compared to women in the higher quartiles (Q_2_ or Q_4_); the odds ratio of SGA was OR = 3.02 (*p* = 0.019) for Q_1_ vs. Q_2_ quartile. The risk profile graph confirms the results. We found that excessive pre-pregnancy BMI (body mass index) affected the estimated SGA odds ratios. Early pregnancy maternal serum selenium status can be a risk marker of SGA newborns and more research is needed in larger groups.

## 1. Introduction

Early identification of women at risk of giving birth to a child with low birth weight is one of the priorities of modern prenatal care. Small-for-gestational age (SGA) birth weight, defined as a weight below the 10th percentile, is associated with increased neonates’ mortality and morbidity, and increased risk of cardiovascular disease in adult life [1]. However, unambiguous risk markers for SGA (<10th percentile) have not yet been established [2,3]. Fetal development is influenced by genetic factors, fetal-placental circulation, environmental factors and maternal nutritional status [4,5,6,7,8] but oxidative stress plays an important role in placental development and placental insufficiency [5,6].

In search of risk markers for birth weight <10th percentile, attention should be paid to selenium due to its involvement in oxidative balance and its importance for human health [9,10,11,12]. Selenium works in the body mainly in the form of selenoproteins, among which there are strong antioxidant enzymes, also present in the placenta (glutathione-GPx peroxidase and Thioredoxin-ThRedx reductase) [5,13]. Along with the normal trophoblast invasion through the spiral artery wall there is an increase in blood flow in the placenta, and this results in an increase in oxidative stress, but antioxidant enzymes protect trophoblast cells [5,6]. It was shown that lower Se levels are associated with lower activity of antioxidant glutathione-GPx peroxidase [5,13]. Importantly, some regions of the world have low selenium status in humans, e.g., in Europe [9,13,14].

In view of the risk of newborn incorrect weight, selenium was assessed in a limited number of studies [11,15,16,17,18]. Mistry et al., in a study consisting of 126 adolescents (14–18 years), found statistically lower maternal plasma Se levels in 28–32 weeks in 19 women giving birth to SGA infants (<10th percentile) vs. 107 women giving birth to appropriate-for-gestational age (AGA) newborns [16]. Bogden et al. found statistical relationships between lower Se concentrations in maternal serum in the 15.6 ± 0.6 week and lower birth weight, in a group of 126 pregnant women who delivered full-term newborns [15]. Choi et al., in a study of 245 pregnant women, found no relationship between lower maternal Se levels in the serum in the first trimester and birth weight below the 10th percentile [11]. In contrast, Wilson et al., in a group of 1065 nulliparous, found a lower risk of SGA (<10th percentile) in a woman with the lowest tertile of plasma Se concentrations in 15 ± 1 week, compared to the highest tertile [17].

Importantly, Se status is influenced not only by geographical and dietary factors, but also by modifiable factors, e.g., BMI (body mass index) [9,19]. It was previously shown that obesity is associated with lower levels of Se [9,19] and higher level of oxidative stress and inflammation [20]. However, it has not yet been investigated whether different maternal BMI categories can affect birth weight odds ratios below the 10th percentile depending on Se concentrations.

The issue whether Se status may be a risk marker of small-for-gestational age (SGA) newborns is open. Therefore, the purpose of this study was to establish the early pregnancy maternal serum selenium concentrations in mothers delivering SGA (<10th percentile) and AGA (between 10–90th percentile) infants and assess the risk of SGA for selenium levels.

## 2. Materials and Methods

The study was approved by the Bioethics Committee of the Medical University of Poznan, Poland (number 769/15). The study was conducted according to the Helsinki Declaration. All the participants signed the informed consent form and the test information form before submitting a blood sample.

### 2.1. Study Population and Method

The pregnant women (*n* = 750) enrolled in this study were recruited at the end of the first trimester of pregnancy at the University Hospital in Poznan, Poland among women taking typical laboratory tests (recruitment in 2015–2016 and observation/analysis in 2017–2018). The hospital is a third-degree reference center with six to eight thousand births per year.

The inclusion criteria included: Healthy white women of Polish descent (Central Europe) from one region (Wielkopolska), aged 18–45 years at conception, in the 10th (+0) − 14th (+6) week of single pregnancy, without aneuploidy, with delivery of a phenotypically normal child ≥25 weeks. The exclusion criteria included: Chronic diseases (apart from being overweight or obesity) as hypertension, diabetes mellitus and thromboembolism, immunological or inflammatory diseases, kidney and liver diseases.

Data (collected from medical records and using a personal questionnaire during the recruitment) included: Obstetrical and gynecological histories, concurrent diseases, medications and supplements, socioeconomic and demographic characteristics, smoking, alcohol consumption, family medical histories (women themselves filled out the questionnaire, but in the presence of midwives); all women declared no alcohol during pregnancy. Pre-pregnancy weight was reported by the participants themselves.

The participants were observed until the 12th week after parturition. We contacted women by telephone or e-mail during pregnancy (they reported complications themselves, e.g., hospitalizations for any reason) and after the postpartum period (after the 12th week after parturition) to obtain information not available in the medical records, e.g., at what gestational age hypertension occurred or whether it receded during the postpartum period; whether smoking ceased or reduced during pregnancy (how many cigarettes were smoked per day); what multivitamin/ microelement preparations were taken in the second to third trimester; whether maternal or newborn complications occurred in the puerperium.

Pregnancy outcomes were taken from the medical records 12 weeks after delivery (the infant sex, gestational age, birth weight of newborns (grams) and diagnosis of intrauterine growth restriction (determined in utero via ultrasound), pregnancy induced hypertension and gestational diabetes mellitus). The gestational age was estimated based on the mother’s last menstrual period and fetal ultrasound measurements. The newborn’s weight was assessed immediately after birth using an automatic/electronic scale. Three groups of newborns were found: SGA, AGA and LGA, based on percentiles of birth weight (small-for-gestational age, appropriate-for-gestational age and large-for-gestational age newborns, respectively).

The estimated minimal sample size was 625 (*p* = 7% for SGA proportion [21], *d* = 2% for margin error and confidence intervals = 95%) using the following formula for a single proportion:

*n* = Z^2^/d^2^ × *p*(1 − *p*) (1)

(“Z = 1.962”—critical value of the normal distribution at α/2, α = 5%).

The base study population (wherein this analysis was nested) constituted 750 women. We excluded 88 women who did not met all inclusion criteria after observation (5 cases of miscarriage, 1 case of delivery before 25th week) and women due to incomplete data (*n* = 82). Among qualified women (*n* = 662), it was found that there were 48 (7.2%) SGA, 548 (82.8%) AGA and 66 (10.0%) LGA newborns.

In this analysis, mothers delivering SGA infants (*n* = 48) and matched mothers delivering AGA infants (*n* = 192) were examined. Small-for-gestational age birth weight (SGA) was defined as a weight below the 10th percentile for gestational age and the gender of the newborn in a given population [22]. Appropriate-for-gestational age birth weight (AGA) was defined as a weight between 10–90th percentile.

### 2.2. Maternal Serum Selenium Determination

Maternal blood samples were taken during recruitment in the 10–14th week of gestation. Processes for obtaining serum and measuring selenium concentrations are described in our previous publication [9]. Maternal sera were stored at −80 °C (until analysis). Before performing analysis maternal sera were thawed, vortexed and centrifuged (at 5000× *g* for 5 min) before selenium determination. The concentrations of the microelements in the maternal serum were determined by ICP (inductively coupled plasma) mass spectrometer: NexION 350D (PerkinElmer, Shelton, CT, USA). Calibration standards were prepared from 10 µg/mL Multi-Element Calibration Standard 3 (PerkinElmer, Shelton, CT, USA) by diluting with blank reagent to the final concentration of 0.1; 0.5; 1.0; 2.0; 5.0; 10 µg/L for measurement of Se; 1, 5, 10 and 50 µg/L for measurement of iron (Fe); 1, 5 and 10 µg/L for measurement of zinc (Zn). Correlation coefficients for calibration curves were always greater than 0.999. General precision was lower than 5% RSD (relative standard deviation). The final concentration included a dilution factor and coefficient which was the mean value of two flanking certified reference material concentrations divided by mean concentration determined by the manufacturer of CRM (certified reference material) [9,10].

### 2.3. Statistical Analyses

The statistical analyses were conducted in the Statistica 13 package. The Shapiro-Wilk test was used for determination of the normality of data distribution. The Mann–Whitney U test was used for continuous variables comparisons (the distributions were not normally distributed). The Pearson’s chi-square test was used for comparisons of categorical variables. In the tests, *p*-value < 0.05 was considered to be significant. The whole cohort and the subgroup (of the normal pre-pregnancy BMI) were equally divided into quartiles, based on the distribution of the maternal selenium (Se) concentrations.

The odds ratios of SGA for Se levels (and 95% confidence intervals CI) were estimated in logistic regression for lower quartiles with regard to the higher quartiles; *p*-value was calculated using the Wald test and *p* < 0.05 was considered to be significant. The odds ratios (OR) were calculated in univariate logistic regression after considering several confounders that were not statistically different between the groups, but were not identical (fetal sex, maternal age, parity, smoking, pre-pregnancy BMI, supplementation of multivitamins/microelements, gestational age at recruitment, gestational weight gain, education level, gestational age at delivery, preeclampsia and gestational diabetes mellitus at present pregnancy). The adjusted odds ratios (AOR) were calculated in multivariate logistic regression after adjusting for confounders that were statistically different between the groups: Maternal height (Model-a). In addition, the second model was calculated (Model-b): After adjusting for maternal height and pack-years in smoking women and pre-pregnancy BMI due to the importance of smoking and BMI for selenium level and SGA risk.

To eliminate the impact of confounding factors on results, we chose the control group (*n* = 192) by individually matching women in the case (SGA) group (*n* = 48) in relation (4:1 ratio) to the following criteria: Maternal age (±2 years) and pre-pregnancy BMI (±10%) and smoking of cigarettes. As confounders, the risk factors for SGA [1,7,8] and related to the concentrations of selenium [9,19] were used.

A graph showing the risk profile of SGA for all selenium concentrations was presented.

## 3. Results

The general characteristics of participants are presented in Table 1. In total, 240 women were examined in this analysis: 48 mothers delivering SGA infants (<10th percentile) and matched 192 mothers delivering AGA infants (10–90th percentile).

The mean age of women in the SGA group was 35.5 years (range 20–45), and in the AGA group was 35.4 years (range 18–45) (*p* = 0.809). The differences between groups were statistically insignificant in terms of fetal sex, maternal age, number of primiparous, number of smokers, pre-pregnancy BMI, supplementation of multivitamins/microelements, gestational age at recruitment, gestational weight gain, education level, gestational age at delivery, preeclampsia and gestational diabetes mellitus at present pregnancy.

The mean maternal serum selenium (Se) concentrations in early pregnancy were lower in women in the SGA group compared to the AGA group (*p* = 0.020) (Table 1).

Complete characteristics of Se concentrations in the groups and subgroups are presented in Table S1. In the whole cohort, the mean Se concentration was 61.95 µg/L (range 41.14–89.17 µg/L) (Table S1). Excessive BMI was associated with lower Se concentrations compared to women with normal pre-pregnancy BMI (60.20 vs. 63.05 µg/L) (*p* = 0.005) (Table S1).

The odds ratios of SGA (<10th percentile) for early pregnancy maternal Se levels are presented in Table 2 and Tables S2 and S3. The highest numbers of SGA were found in the lowest Q_1_ quartile of Se.

In the whole cohort, women in the lowest Q_1_ quartile (≤56.60µg/L) had a 2.63-fold increase in SGA risk compared to women in Q_4_ quartile (OR = 2.63; *p* = 0.034). The results were higher after excluding the excessive BMI (Table 2).

The results were sustained after adjusted for maternal height (Table S2). The odds ratios of SGA for the lowest Se concentrations (in Q_1_) compared to Q_2,_ Q_3_ and Q_4_ quartiles are presented in Table S3. Women in the lowest Q_1_ quartile had a 3.02-fold increase in SGA risk compared to Q_2_ quartile (*p* = 0.019) (Table S2). The results were sustained after adjusted for maternal height, pre-pregnancy BMI and pack-years in smoking women (Table S3).

**Figure 1:**
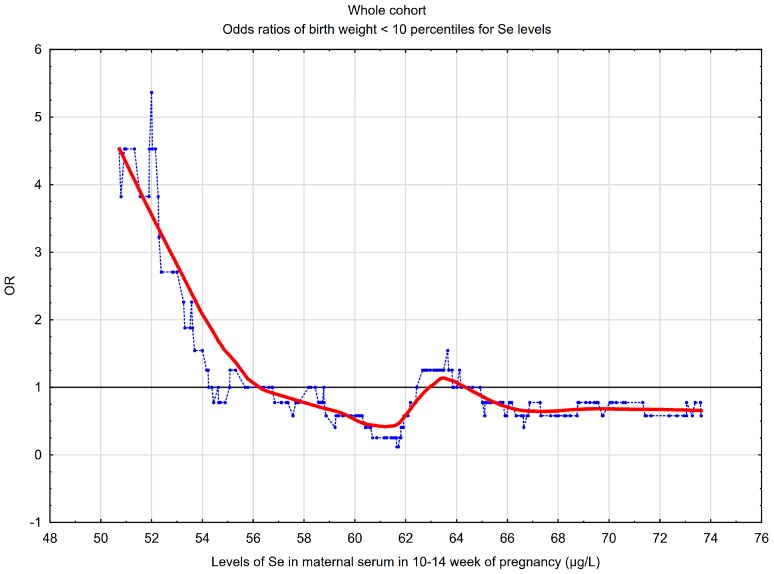
The risk of SGA newborns (<10th percentile) for maternal serum selenium (Se) concentrations in the 10–14th week of pregnancy, in the whole cohort (*n* = 240). The graph shows the changes in the odds ratio (OR) of SGA, calculated on a sliding window with respect to the changes in the selenium concentrations. The window width adopted was 30 observations. The points (blue) correspond to the odds ratios of SGA in a window containing a fixed number of neighboring cases (the center of the window is for a given Se concentration value). The curve (red) represents the SGA risk profile smoothed with the Lowess method. The horizontal line (black) marks the reference line for OR = 1; the points above the line indicate an increased risk (OR >1).

A graphical picture of the SGA risk profile for selenium concentrations is presented in Figure 1. The graph confirms the results obtained between quartiles. The lowest concentrations of early pregnancy maternal serum Se were associated with a higher risk of SGA. In the whole cohort, the threshold point was 56.1 µg/L, below which a “steep” increase in the SGA risk was observed.

## 4. Discussion

The results of this study showed that maternal serum selenium in the 10–14th week was a risk marker for small-for-gestational age birth weight (SGA), defined as a weight below 10th percentile for gestational age and the gender of the newborn. The mean maternal Se concentrations were lower in mothers delivering SGA newborns compared to mothers delivering AGA newborns (between 10–90th percentile) (59.60 vs. 62.54 µg/L; *p* = 0.020). Women with the lowest levels of Se (in quartile Q_1_) had about three times higher risk of SGA compared to women with higher Se levels (in quartiles Q_2_, Q_3_ or Q_4_). The risk profile graph (in Figure 1) confirms and highlights these results. Our results were obtained after taking into account many confounders. Excessive pre-pregnancy BMI affected the estimated SGA odds ratios.

When analyzing the literature, one can conclude that some studies confirm our results. Prospective relationships between lower maternal Se levels and lower birth weight were obtained by Bogden et al. (for serum Se concentrations in ±15.6 week) and by Mistry et al. (for plasma Se concentrations in weeks 28–34) [15,16]. Tsuzuki et al. showed a relationship between low birth weight and lower serum selenium concentrations in the mother before delivery and in the child (after delivery) [23]. These relationships were not confirmed by other authors [11,17,18]. Concerning the reasons for the discrepancies, we found, among other factors, population heterogeneity, lower sizes of studied groups and different measurement methods.

Several factors may affect selenium levels. Genetic conditioning and geographical differences, diet, supplementation, age, gender, BMI, smoking, and health affect the selenium status of the body [9,11,14,19]. The content of this element in the diet is related to geographical location and its content in the soil [14]. During pregnancy, there is a decrease in the level of selenium in subsequent trimesters, although the processes associated with it are not explained [11].

Small-for-gestational age birth weight (<10th percentile) is a heterogenous group, including constitutionally small fetuses (caused by family and genetic factors) and newborns with intrauterine growth restriction (caused by placental insufficiency) and small newborns (caused by factors other than insufficiency of the placenta) [3,5]. At the same time, we point out that fetuses with intrauterine growth restriction are pathologically small irrespective of the centile of growth (even if they fall into the <10th percentile category or in the 10–90th percentile category) [24]. Previously, birth weight was linked with inter alia, genetic factors and fetal-placental circulation, fetal gender, gestational age, parity, the mother’s age and mother’s height, nutritional status, BMI and gestational weight gain, vitamin/micronutrient supplementation, smoking and concomitant diseases in pregnancy (e.g., preeclampsia and gestational diabetes mellitus) [1,7,8,25]. In our study, the influence of many confounders was excluded by matching maternal characteristics between groups.

Firstly, our study provided evidence of an interesting relationship between lower maternal serum Se levels in the 10–14th week and small-for-gestational age birth weight (<10th percentile). The mechanisms of these relationships remain unclear. However, the deficiency of antioxidant activities of selenium during placenta development has been taken into consideration [5,6]. The development of the placenta circulatory system is crucial in embryo development and fetus growth [5,6]. It was shown that lower selenium levels are associated with lower activity of antioxidant glutathione-GPx peroxidase, which is required to protect the placenta and fetus from oxidative stress [5,13]. However, Se is involved in numerous biochemical/biological processes (inter alia, the oxidative balance, inflammatory and immune processes and apoptosis) that are part of the complex processes of early placental development [13]. Selenium’s role in heavy metal detoxification has also been taken into consideration [26,27].

Secondly, our study showed that women with excessive pre-pregnancy BMI had significantly lower early pregnancy serum Se levels, compared to women with normal BMI (*p* = 0.006), which is in line with literature reports [9,19]. This influenced the small-for-gestational age newborns’ (<10th percentile) odds ratio values. We obtained higher odds ratios in the subgroup of women with normal pre-pregnancy BMI than in the entire cohort: OR = 4.27 (*p* = 0.015) and OR = 2.63 (*p* = 0.034), respectively, for Se concentrations in the lowest Q_1_ quartile compared to the highest Q_4_ quartile. Our results suggest that the structure of the studied population in terms of BMI may be an important reason for discrepancies between studies.

The main clinical implications of our study are related to the usefulness of the simple measurement of maternal selenium concentrations in early pregnancy in predicting small-for-gestational age birth weight (<10th percentile). In our study, the threshold point was 56.1 µg/L, below which a “steep” increase in SGA risk was observed. Our results may suggest the need to optimize Se levels in pre-pregnancy and in the early pregnancy period, and especially in women with pre-existing obesity/overweight. Our results may suggest the need to optimize maternal pre-pregnancy weight. Recommendations for the optimal amount of Se in the diet are based on maximizing glutathione peroxidase activity [28]. The optimal average serum selenium concentration—according to the World Health Organization—is 39.5–194.5 µg/L for healthy adults, while concentrations of 70–90 µg/L were found to maximize glutathione-GPx peroxidase activity [28]. The average demand was estimated at 60 µg/day for pregnant women.

This is important in light of the selenium deficiency in humans found in some regions of the world, e.g., in Europe where the average Se serum/plasma concentrations in poles are 50–55 µg/L [9,14]. Worldwide and national interventions are undertaken to supplement the deficiencies by enriching diets with this trace element [14]. Recent research takes into account the differences between organic Se supplements (containing selenomethionine, of higher bioavailability) and inorganic supplements (selenites and selenates). However, selenium is a trace element and both its deficiency and excess leads to disorders [12,14]. Therefore, supplementation of this element should be carried out only in persons with confirmed deficiency [29].

We are aware of the complexity of the processes accompanying fetal development and the involvement of numerous biomarkers and microelements. In this study, we also presented the average concentrations of other micronutrients involved in the oxidative balance (zinc and iron) to show that their concentrations did not differ statistically between groups, but this issue was not analyzed in this study. Our results suggest the need for further research on mechanisms that link maternal selenium levels to fetal development.

### Advantages and Limitations

The main strength of this study was the assessment of maternal selenium levels in early pregnancy and an analysis of the prospective cohort of pregnant women. Our study was conducted in well-matched groups. Matching many maternal features between the study groups allowed the impact of many confounding factors to be excluded. We separately assessed the women in the whole cohort and in the subgroup of normal pre-pregnancy BMI showing the impact of BMI on estimated risk.

There are, however, a few limitations. A larger sample would allow for an analysis in several subgroups. Early fetal development processes are multifaceted and complex, and other interfering factors may exist, which is why studies with more cases are needed. The insufficient number of studies assessing the potential mechanisms of the association of Se with birth weight makes the interpretation of the results more difficult. An additional assessment of selenium levels at other time points could be interesting.

## 5. Conclusions

In our study, we found that maternal serum selenium levels in the 10–14th week were lower in women who gave birth to infants that were small-for-gestational age (<10th percentile) than in women who gave birth to newborns appropriate-for-gestational age in this respect (between 10–90th percentile). Considering many confounders, we found a risk of birthweight below the 10th percentile to be about three times higher in the women with the lowest selenium concentrations (in Q_1_ quartiles) compared to the women with higher concentrations (in Q_2_–Q_4_ quartiles).

Our results suggest that selenium in the maternal serum in early pregnancy may be a marker of SGA newborns (<10th percentile) risk. The simple measurement of maternal serum Se concentrations would allow for early identification of the women at risk of such a pregnancy outcome. However, more research is needed in larger groups.

We also found a relationship between an excessive pre-pregnancy BMI and lower selenium concentrations, which affected the estimated SGA birthweight (<10th percentile) odds ratios. These results highlight the structure of the studied population in terms of various BMI categories as the cause of the discrepancies between studies.

Our results may also suggest the need to optimize selenium levels in women before pregnancy and in early pregnancy, especially in the women with pre-existing overweight and obesity, but this requires well-designed randomized studies.

Our results may suggest the need to optimize maternal pre-pregnancy weight.

## Figures and Tables

**Table 1:** The characteristics of women in the AGA and SGA group.

	Controls (AGA Group) (*n* = 192)	Cases (SGA Group) (*n* = 48)	
Characteristics	Mean (SD) or *n* (%)	Mean (SD) or *n* (%)	*p* *
Maternal age (years)	35.4 (4.7)	35.5 (4.7)	0.809
Maternal age (range)	(18–45)	(20–45)	
Primiparous	78 (40.6%)	19 (39.6%)	0.895
Fetal hypotrophy in history	2 (1.0%)	5 (10.4%)	0.004
Gestational age at recruitment (weeks)	12.1 (0.8)	12.0 (0.9)	0.454
Pre-pregnancy BMI (kg/m²)	25.3 (4.6)	24.9 (4.8)	0.556
Pre-pregnancy BMI (range)	(17.7–40.0)	(17.2–37.9)	
Pre-pregnancy BMI ≥30 kg/m²	32 (16.7%)	7 (14.6%)	0.900
Normal pre-pregnancy BMI (18.5–24.9 kg/m²)	28 (58.5%)	106 (55.2%)	0.697
Maternal height (cm)	166.4 (6.6)	163.9 (7.2)	0.024
Women who have never smoked	153 (79.7%)	34 (70.8%)	0.186
Folic acid in I trimester **	64 (33.3%)	13 (27.1%)	0.407
Multivitamins in II–III trimester **	89 (46.4%)	23 (47.9%)	0.846
Education <12 years (for available data)	13 (8.0%)	7 (18.9%)	0.065
**Outcomes**			
Fetal sex/son	100 (52.1%)	25 (52.1%)	1.000
Newborn birth weight (g)	3302.5 (486.5)	2358.0 (511.1)	<0.0001
IUGR	2 (1.0%)	8 (16.7%)	0.0001
Gestational age at delivery (weeks)	38.5 (1.9)	37.9 (2.6)	0.100
APGAR-1′ <7 ***	7 (3.7%)	2 (4.1%)	1.000
Preeclampsia	5 (2.6%)	3 (6.3%)	0.200
Gestational diabetes mellitus	39 (20.3%)	10 (20.8%)	0.936
Microelements ** **			
Selenium (µg/L)	62.54 (7.50)	59.60 (8.60)	0.020
Zinc (µg/L)	628.77 (209.72)	631.09 (97.19)	0.258
Iron (µg/L)	1059.34 (349.12)	969.28 (301.28)	0.130

* The Mann–Whitney U test was used for comparisons of continuous variables and medians were compared, and the Pearson chi-square test was used for categorical variables comparisons (*p*-value < 0.05 was assumed to be significant); ** multivitamin/microelement preparations; *** APGAR-5′ < 7 was not found; ** ** microelements were measured in serum from the 10–14th week; IUGR: intrauterine growth restriction; AGA: appropriate-for-gestational age newborns (10–90th percentile); SGA: small-for-gestational age newborns (<10th percentile).

**Table 2:** The odds ratios of small-for-gestational-age newborns (SGA) for early pregnancy maternal serum selenium levels, in logistic regression.

Quartile	Selenium (µg/L) !	Odds Ratios of Small–for–Gestational Age (SGA) Newborns		
		SGA (Cases)	AGA (Controls)	OR * (95% CI:); *p* **
**Whole Cohort (*n* = 240)**				
Q_1_	41.14–56.60	19	41	2.63 (1.08–6.42); 0.034
Q_2_	56.60–61.86	8	52	0.87 (0.36–2.14); 0.794
Q_3_	61.86–66.62	12	48	1.42 (0.55–3.66); 0.472
Q_4_	66.62–89.17	9	51	1
**Subgroup Normal BMI # (*n* = 134)**				
Q_1_	41.14–58.27	14	19	4.27 (1.32–13.82); 0.015
Q_2_	58.27–62.86	3	31	0.56 (0.12–2.55); 0.456
Q_3_	62.86–67.69	6	27	1.29 (0.35–4.70); 0.701
Q_4_	67.69–89.17	5	29	1

! Serum selenium concentrations were measured in the 10–14th week and border values were included in lower quartile; # Pre-pregnancy body mass index 18.50–24.99 kg/m2; * OR: crude odds ratio calculated in univariate logistic regression (after matching confounders); ** *p*-value obtained using the Wald test, *p* < 0.05 was assumed to be significant CI: confidence intervals; AGA: 10–90th percentile of birth weight; SGA: <10th percentile of birth weight.

## Supplementary Materials

The following are available online at https://www.mdpi.com/2072-6643/11/10/2298/s1, Table S1: The complementary characteristics of early pregnancy maternal serum selenium concentrations in the SGA and AGA group, Table S2: The odds ratios of small-for-gestational age newborns (SGA) for early pregnancy maternal serum selenium levels, in multivariate logistic regression (Model-a), Table S3. The odds ratios of small-for-gestational-age newborns (<10th percentile) for early pregnancy maternal serum selenium levels, in multivariate logistic regression (Model-b).
