# Supplementary material for: The Role of Early Pregnancy Maternal Selenium Levels on the Risk for Small-for-Gestational Age Newborns
Source: Nutrients. 2019 Sep 26;11(10):2298. doi: 10.3390/nu11102298 (PMC6836167; doi:10.3390/nu11102298)
Supplement: Supplementary file 1 [file nutrients-11-02298-s001.pdf]

Table 1. The complementary characteristics of early pregnancy maternal serum selenium concentrations in the SGA and AGA group.

Groups	n	Characteristics of selenium (Se) concentrations *			
		Mean	Range	Median	p **
**Whole cohort**	240	61.951	41.140 – 89.173	61.883	
Controls (AGA) •	192	62.540	44.388 – 89.173	61.941	0.020
Cases (SGA) •	48	59.598	41.140 – 81.402	59.367	
**Pre-pregnancy BMI categories**					
BMI ≥ 25 kg/m^2	100	60.204	44.388 – 82.462	59.532	0.005
BMI 18.5-24.99 kg/m^2	134	63.045	41.140 – 89.173	62.911	
**Subgroup of normal BMI** #					
Controls (AGA) •	106	64.100	45.128 – 89.173	63.229	0.006
Cases (SGA) •	28	59.048	41.140 – 81.402	58.839	

* Selenium concentrations were measured in serum from the 10-14th gestational week; **p- value obtained using the Mann-Whitney U test ($p < 0.05$ was assumed to be significant); # normal body mass index: 18.5-24.99 kg/m^2; AGA: birth weight between 10-90th percentile; SGA: birth weight <10th percentile.

Table 2. The odds ratios of small-for-gestational-age newborns (SGA) for early pregnancy maternal serum selenium levels, in multivariate logistic regression (Model-a).

Quartile	Selenium (µg/L) !	Odds ratios of small-for-gestational age (SGA) newborns	
		OR * (95% CI:); p **	AOR-a * (95% CI:); p **
**Whole cohort**			
Q1	41.14-56.60	2.63 (1.08-6.42); 0.034	2.41 (0.97-5.94); 0.050
Q2	56.60-61.86	0.87 (0.36-2.14); 0.794	0.84 (0.30 -2.38). 0.747
Q3	61.86-66.62	1.42 (0.55-3.66); 0.472	1.37 (0.53-3.56); 0.519
Q4	66.62-89.17	1	1

! Serum selenium concentrations were measured in the 10-14 week and border values were included in the lower quartile; *OR: crude odds ratios calculated in univariate logistic regression (after matching confounders) and AOR-a: adjusted odds ratio calculated in multivariate logistic regression (after adjusted for maternal height); ** p- value obtained using the Wald test ($p < 0.05$ was considered to be significant); CI: confidence intervals.

Table S3. The odds ratios of small-for-gestational-age newborns (<10th percentile) for early pregnancy maternal serum selenium levels, in multivariate logistic regression (Model-b)

Quartile	Se (µg/L) !	Odds ratios of small-for-gestational age (<10th percentile) newborns	
		* OR (95% CI:); p **	* AOR-b (95% CI:); p **
**Whole cohort (N=240)**			
Q1	41.14-56.60	3.02 (1.20-7.57); 0.019	3.95 (1.36-11.54); 0.012
Q2	56.60-61.86	1	1
Q1	41.14-56.60	1.85 (0.81-4.27); 0.147	1.74 (0.67-4.57); 0.257
Q3	61.86-66.62	1	1
Q1	41.14-56.60	2.63 (1.08-6.42); 0.034	2.33 (0.87-6.28); 0.094
Q4	66.62-89.17	1	1

! Serum Se concentrations were measured in the 10-14th week and border values were included in lower quartile; *OR: crude odds ratios calculated in univariate logistic regression (after matching confounders) and AOR-b: adjusted odds ratios after adjusted for maternal height <160cm, pre-pregnancy BMI and pack-years in smoking women; **p- value obtained using the Wald test ($p < 0.05$ was considered to be significant); CI: confidence intervals.
